# Clinicopathologic implication of PD-L1 and phosphorylated STAT3 expression in diffuse large B cell lymphoma

**DOI:** 10.1186/s12967-018-1689-y

**Published:** 2018-11-20

**Authors:** Hyun Jung Kwon, Jeong Mi Yang, Jeong-Ok Lee, Jong Seok Lee, Jin Ho Paik

**Affiliations:** 1 Department of Pathology, Seoul National University Bundang Hospital, Seoul National University College of Medicine, 300 Gumi-dong, Bundang-gu, Seongnam, 463-707 South Korea; 2 Department of Internal Medicine, Seoul National University Bundang Hospital, Seoul National University College of Medicine, Seongnam, South Korea

**Keywords:** Diffuse large B cell lymphoma, PD-L1, pSTAT3, Microenvironment, Prognosis

## Abstract

**Background:**

Antitumor immune response of programmed cell death ligand (PD-L1) has shown clinical value not only in Hodgkin lymphoma and EBV-associated lymphomas but also in EBV-negative diffuse large B cell lymphoma (DLBCL) of non-germinal center B cell-like (non-GCB) subtype. Signal transducer and activator of transcription 3 (STAT3) is known to induce PD-L1 in immune cells and its activated form, phosphorylated STAT3 (pSTAT3), is also frequently expressed in non-GCB DLBCL. Herein, we investigated associations between PD-L1 expression/gene alteration, pSTAT3 expression and clinicopathologic variables in EBV-negative DLBCL.

**Methods:**

In 107 cases of DLBCLs with non-GCB subtype (67%; 72/107), GCB subtype (25%; 27/107) and unclassifiable cases (8%; 8/107), we performed PD-L1 and pSTAT3 immunohistochemistry and fluorescence in situ hybridization for PD-L1 gene translocation and copy number gain/amplification.

**Results:**

PD-L1 was expressed in tumor cells (PD-L1t) in 21% (23/107; 30% cutoff), immune cells (PD-L1i) in 36% (38/107; 20% cutoff), and pSTAT3 in tumor nuclei in 41% (44/107; 40% cutoff). PD-L1 gene alteration was observed in 10% (10/102) including translocation in 6% (6/102) and copy number gain/amplification in 4% (4/102). Non-GCB subtype was associated with PD-L1t and pSTAT3 (p = 0.006 and p = 0.042), and tended to have PD-L1 gene alteration (p = 0.058). Tumoral PD-L1 expression without gene alteration (PD-L1t+ GA−) correlated with pSTAT3-positive tumor cell proportions (%) (p = 0.033). In survival analysis, pSTAT3 expression independently predicted shorter PFS in total cohort (p = 0.017) and R-CHOP-treated group (p = 0.007), and in pSTAT3-negative R-CHOP-treated subset, PD-L1 expression in immune cells (PD-L1i) correlated with shorter PFS (p = 0.042).

**Conclusions:**

Gene alteration and protein expression of PD-L1 and pSTAT3 expression were closely related in DLBCL and constituted features of non-GCB subtype. In addition to known clinical significance of pSTAT3, immune cell expression of PD-L1 (PD-L1i) had also clinical value in pSTAT3-dependent manner. These findings may provide an insight into immunotherapeutic strategy and risk stratification in DLBCL patients.

**Electronic supplementary material:**

The online version of this article (10.1186/s12967-018-1689-y) contains supplementary material, which is available to authorized users.

## Background

Immune escape and immune suppression has emerged as important mechanisms for the development of solid and hematologic malignancies. Research on immunotherapy has thus opened a new era in the treatment of many malignancies. Recently, impressive advancements in the treatment of hematologic malignancies with the immune checkpoint inhibitors targeting programmed cell death-1 (PD-1) receptor and its ligand (PD-L1) lead to the approval of nivolumab and pembrolizumab by the Food and Drug Administration for the treatment of classical Hodgkin lymphoma in relapsed or refractory cases.

Diffuse large B-cell lymphoma (DLBCL) is the most common type of non-Hodgkin lymphoma in adults. The 5-year overall survival reaches only 60–70% when treated with standard chemotherapy of rituximab plus cyclophosphamide, doxorubicin, vincristine and prednisone (R-CHOP) [1]. DLBCL is known to be heterogeneous in genetic and prognostic aspects. The subclassification of DLBCL based on cell-of-origin using the Hans algorithm proved useful in predicting prognosis as germinal center B-cell like (GCB) subtypes have better outcomes than non-GCB subtypes [2, 3].

PD-L1 research on DLBCL has found that not only tumor B-cells but also non-malignant immune cells in the microenvironment express PD-L1 [4–7]. In addition, many studies have reported that non-GCB DLBCL shows higher PD-L1 expression than GCB DLBCL [5, 8, 9], and that similar to Hodgkin lymphoma, Epstein–Barr virus (EBV) infection probably plays an important role in inducing PD-L1 expression in DLBCL [4, 10, 11].

The mechanisms of PD-L1 expression in DLBCL has not been elucidated clearly. In many subtypes of lymphoma, including classical Hodgkin lymphoma, primary mediastinal B-cell lymphoma and primary central nervous system lymphoma, genetic alterations such as copy-number alterations, amplification, and translocation were observed on the 24.1 locus of the short arm of chromosome 9. On this locus lie the genes encoding PD-L1, PD-L2 and JAK2, resulting in overexpression of PD-L1 when such genetic alterations occur [7, 11, 12]. In DLBCL, however, only a subgroup of less than 20% has these genetic changes, most of which are primary mediastinal large B cell lymphoma [8, 13]. Thus, other mechanisms are currently being investigated.

Infection by EBV upregulates PD-L1 expression in tumor cells either directly by binding to latent membrane protein (LMP-1) that triggers a signaling cascade involving JUN-B or indirectly by activating inflammatory cytokines [14]. Inflammatory cytokines, represented by IFN-γ and IL-10, in turn induce PD-L1 expression via the Janus kinase (JAK)/signal transducer and activator of transcription (STAT) signaling pathway in Hodgkin and non-Hodgkin lymphoma. Other mechanisms also involve the JAK/STAT signaling pathway such as inhibition of Suppressor of cytokine signaling-1 (SOCS-1) or microRNA miR-135a [7].

Phosphorylated STAT3 (pSTAT3), a transcription factor, is a key player of the JAK/STAT pathway that regulates cell proliferation and survival [15]. Cytoplasmic protein STAT3 moves into the nucleus and binds to the promoter of PD-L1 gene when phosphorylated by JAK to enhance transcription [16, 17]. This process can be stimulated by LMP-1 of EBV, resulting in concomitant overexpression of pSTAT3 with increased PD-L1 expression in nasopharyngeal carcinoma [14]. Reed-Sternberg cells stain positive for both PD-L1 and pSTAT3, suggesting an association between these two markers in lymphoma [18]. A few reports discovered association of PD-L1 and pSTAT3 expression in natural killer/T cell lymphoma [17, 19], ALK-negative anaplastic large cell lymphoma [20] and cell lines of adult T cell leukemia/lymphoma [21]. In DLBCL, pSTAT3 is overexpressed, similar to PD-L1, in non-GCB subtype and shows higher expression than GCB subtypes [22–28]. The association between PD-L1 and pSTAT3 in DLBCL, however, has not yet been explored.

In this study, we evaluated the protein expression of PD-L1 in terms of its potential intrinsic and extrinsic mechanism, i.e., PD-L1 gene alteration and STAT3 activation, in EBV-negative DLBCLs. We also investigated the clinical significance of PD-L1/pSTAT3-related biomarkers in association with clinicopathologic parameters and prognosis in DLBCL patients.

## Methods

### Patients and samples

Our cohort consisted of 107 patients newly diagnosed with de novo DLBCL, not otherwise specified (NOS) between May 2005 and January 2013 at Seoul National University Bundang Hospital. Clinicopathological information was obtained from clinical records and pathology reports. Large B cell lymphomas with distinct entities including ‘EBV-positive DLBCL, NOS’, ‘DLBCL of the central nervous system’, ‘primary mediastinal large B cell lymphoma’ as well as transformed DLBCL from low grade lymphomas were excluded. The pathologic classification was based on the 2016 Revised 4th edition of World Health Organization classification [29] and further subtyped by Hans algorithm [2] by experienced hematopathologists. The study protocol was approved by the Institutional Review Board of Seoul National University Bundang Hospital.

### Construction of tissue microarray (TMA)

Hematoxylin and eosin (H & E)-stained slides were reviewed in each case to confirm the original diagnosis and select the most representative sections. A tissue microarray (TMA) was constructed using 2 mm-diameter cores derived from the representative areas of the formalin-fixed paraffin-embedded tissue blocks from each case and from normal tonsils for controls by SuperBioChips Laboratories (Seoul, Korea), as previously described [30].

### Immunohistochemical staining

TMAs were sectioned at a thickness of 4-μm and stained with Benchmark XT and Benchmark ULTRA (Roche Diagnostics) along with normal tonsil specimens as controls for the following antibodies (clones): anti-Bcl-2 (std32, 1:50, Dako), anti-Bcl-6 (std32, ready-to-use, Ventana), anti-CD10 (std32, ready-to-use, Ventana), anti-Mum-1 (mild32, 1:150, Dako), anti-PD-L1 (E1L3N, 1:100, Cell Signaling) and anti-pSTAT3 (#9131, 1:20, Cell Signaling). Using immunochemistry results, the Hans algorithm was applied to each case as described previously [31].

### Evaluation of PD-L1 and pSTAT3 immunohistochemistry

The immunohistochemistry slides were scored by two pathologists (H.J.K and J.H.P). PD-L1 was scored both in tumor B-cells and in non-malignant immune cells. PD-L1 was considered positively expressed in tumor or non-malignant immune cells if membranous staining alone or membranous and cytoplasmic staining together was present. The percentage of stained tumor B-cells and non-malignant immune cells were estimated in each TMA core regardless of intensity. Tumor cells were distinguished from non-malignant immune cells by histologic clues such as nuclear enlargement and atypism, followed by comprehensive interpretation with other immunohistochemical markers including CD20, BCL2, BCL6, CD10 and MUM1. Since an optimal cut-off could not be determined by receiver operating characteristic (ROC) curve analysis, cases were classified by a 30% cutoff for tumor B-cells and 20% cutoff for non-malignant immune cells according to previous studies [6]. pSTAT3 expression was scored in tumor B-cells alone with cutoff value of 40%, which was set based on ROC curve analysis. In addition, the proportion (%) of pSTAT3-positive cells was digitally counted by using digital slide scanner and image analyzer (3DHISTECH, Budapest, Hungary) for correlation analysis.

### Fluorescence in situ hybridization for PD-L1 gene

For fluorescence in situ hybridization (FISH) staining, PD-L1 break-apart probe (9p24.1) (catalog # PDL1BA-20-ORGR) and PD-L1 (orange)/chromosome 9 (green) probe set (9p24.1/9p21.33) (catalog # PDL1-CHR09-20-ORGR) were purchased from empire genomics (Buffalo, NY). The FISH staining process was performed as previously described [32]. Briefly, after deparaffinization and dehydration, slides were immersed in 2 M HCl, boiled using a microwave oven in citrate buffer (pH 6.0), incubated in 1 M NaSCN for 40 min at 80 °C, immersed in a protease buffer and fixed in 10% neutral buffered formalin. After applying the DNA probe set to the slides, they were incubated in a humidified chamber at 83 °C for 3 min to denature the target DNA and probe, and subsequently incubated overnight at 37 °C to achieve hybridization. Following post-hybridization washing, 4,6-diamidino-2-phenylindole (DAPI) and an anti-fade compound (*p*-phenylenediamine) were applied to the slides as a counter-stain. An Olympus BX51TRF microscope (Olympus Corp., Tokyo, Japan) equipped with the appropriate filter sets was used to analyze the stained cells. For the interpretation of translocation and/or copy number gain/amplification, more than 200 tumor cells in non-overlapping intact nuclei were counted [17, 32]. Separation of orange and green signals in more than 15% of tumor cells were interpreted as translocation [31]. Copy number gain and amplification were defined as PD-L1 gene/chromosome 9 ratio > 2 and > 4, respectively.

### Statistical analysis

Statistical analysis was carried out using SPSS Statistics ver. 19 (IBM, Chicago, IL). Chi square or Fisher’s exact test was used to compare clinicopathological characteristics. Pearson’s correlation test was performed to determine the association of immunohistochemical expressions. Progression-free survival (PFS) was defined as the time interval from the start date of treatment to the date of progression including radiologically confirmed progressive disease based on positron-emission tomography (PET)-computed tomography (CT) or CT, refractoriness to the first-line therapy in response evaluation after second cycle or completion of treatment, relapse or death. Overall survival (OS) was defined as the time interval from the date of diagnosis to the date of last follow-up or death. The Kaplan–Meier analysis was used to construct survival curves, with which the log-rank test compared the difference. A multivariate analysis by Cox proportional hazards regression modeling was performed. All *p* values reported are two-sided and statistical significance was accepted with those less than 0.05.

## Results

### Clinicopathologic characteristics

The characteristics of 107 patients with DLBCL are summarized on Table 1. Briefly, our cohort consisted predominantly of non-GCB subtype (67%; 72/107) compared to GCB subtype (25%; 27/107) or unclassifiable cases (8%; 8/107) by Hans algorithm. The total cohort mainly included cases with good Eastern Cooperative Oncology Group performance status (ECOG PS) (< 2; 91%; 97/107), absence of B symptoms (79%; 85/107), low international prognostic index (IPI; 66%; 71/107), less than 2 extranodal site involvements (76%; 81/107), absence of bone marrow involvement (79%; 84/107) and non-bulky masses (92%; 98/107). Most of the patients received R-CHOP chemotherapy (87%; 93/107). Compared to the patients with GCB subtype, the patients with non-GCB subtype frequently had elevated serum lactate dehydrogenase levels (p = 0.040). Non-GCB subtype also tended to be associated with a high IPI score (3–5; p = 0.056) and presence of B symptoms (p = 0.088), which did not reach statistical significance.

**Table 1:** Clinicopathologic characteristics according to Hans classification in diffuse large B cell lymphoma patients

Clinicopathologic characteristics	Total	GCB	Non-GCB	p-value
Age				
≤ 60	53 (50%)	17 (63%)	31 (43%)	0.078^#^
> 60	54 (50%)	10 (37%)	41 (57%)	
Sex				
Male	61 (57%)	7 (26%)	34 (47%)	0.055^#^
Female	46 (43%)	20 (74%)	38 (53%)	
Primary site				
Nodal	54 (50%)	23 (85%)	51 (71%)	0.196^†^
Extranodal	53 (49%)	4 (15%)	21 (29%)	
B symptoms				
Absent	85 (79%)	25 (93%)	55 (76%)	0.088^†^
Present	22 (21%)	2 (7%)	17 (24%)	
ECOG PS				
< 2	97 (91%)	26 (96%)	64 (89%)	0.437^†^
≥ 2	10 (9%)	1 (4%)	8 (11%)	
Serum lactate dehydrogenase				
Normal	54 (50%)	18 (69%)	32 (46%)	0.040^#^
Elevated	50 (47%)	8 (31%)	38 (54%)	
Unknown	3 (3%)			
No. of extranodal sites				
< 2	81 (76%)	17 (63%)	33 (46%)	0.129^#^
≥ 2	26 (24%)	10 (37%)	39 (54%)	
Ann Arbor stage				
I–II	56 (52%)	17 (63%)	35 (49%)	0.203^†^
III–IV	51 (48%)	10 (37%)	37 (51%)	
International prognostic index				
0–2	71 (66%)	22 (81%)	44 (61%)	0.056^#^
3–5	36 (34%)	5 (19%)	28 (39%)	
Bone marrow involvement				
Absent	84 (79%)	25 (96%)	55 (85%)	0.168^†^
Present	14 (13%)	1 (4%)	10 (15%)	
Unknown	9 (8%)			
Bulky mass (cm)				
< 10	98 (92%)	25 (93%)	66 (92%)	> 0.999^†^
≥10	9 (8%)	2 (7%)	6 (8%)	
Hans classification				
GCB	27 (25%)	27 (100%)	0 (0%)	NA
Non-GCB	72 (67%)	0 (0%)	72 (100%)	
Unclassifiable	8 (8%)			
BCL2 expression				
Negative	42 (39%)	13 (50%)	24 (33%)	0.133^#^
Positive	65 (61%)	13 (50%)	48 (67%)	
BCL6 expression				
Negative	60 (56%)	8 (30%)	44 (61%)	0.005^#^
Positive	47 (44%)	19 (70%)	28 (39%)	
CD10 expression				
Negative	92 (86%)	12 (44%)	72 (100%)	< 0.001^†^
Positive	15 (14%)	15 (56%)	0 (0%)	
MUM1 expression				
Negative	41 (38%)	25 (93%)	8 (11%)	< 0.001^†^
Positive	66 (62%)	2 (7%)	64 (89%)	
Treatment regimen				
R-CHOP	93 (87%)	23 (85%)	64 (89%)	0.731^†^
Others	14 (13%)	4 (15%)	8 (11%)	
Total N (%)	107 (100%)			

NA: not applicable; ECOG PS: the Eastern Cooperative Group Performance Status; GCB: germinal center B-cell like; R-CHOP: rituximab with cyclophosphamide–doxorubicin–vincristine–prednisone, GI: gastrointestinal

p values were calculated by using Fisher’s exact test (2-sided)^†^ or Pearson’s Chi square test (2-sided)^#^

### Association between clinicopathologic variables and protein expression and gene alteration of PD-L1 and pSTAT3 expression

**Fig. 1:**
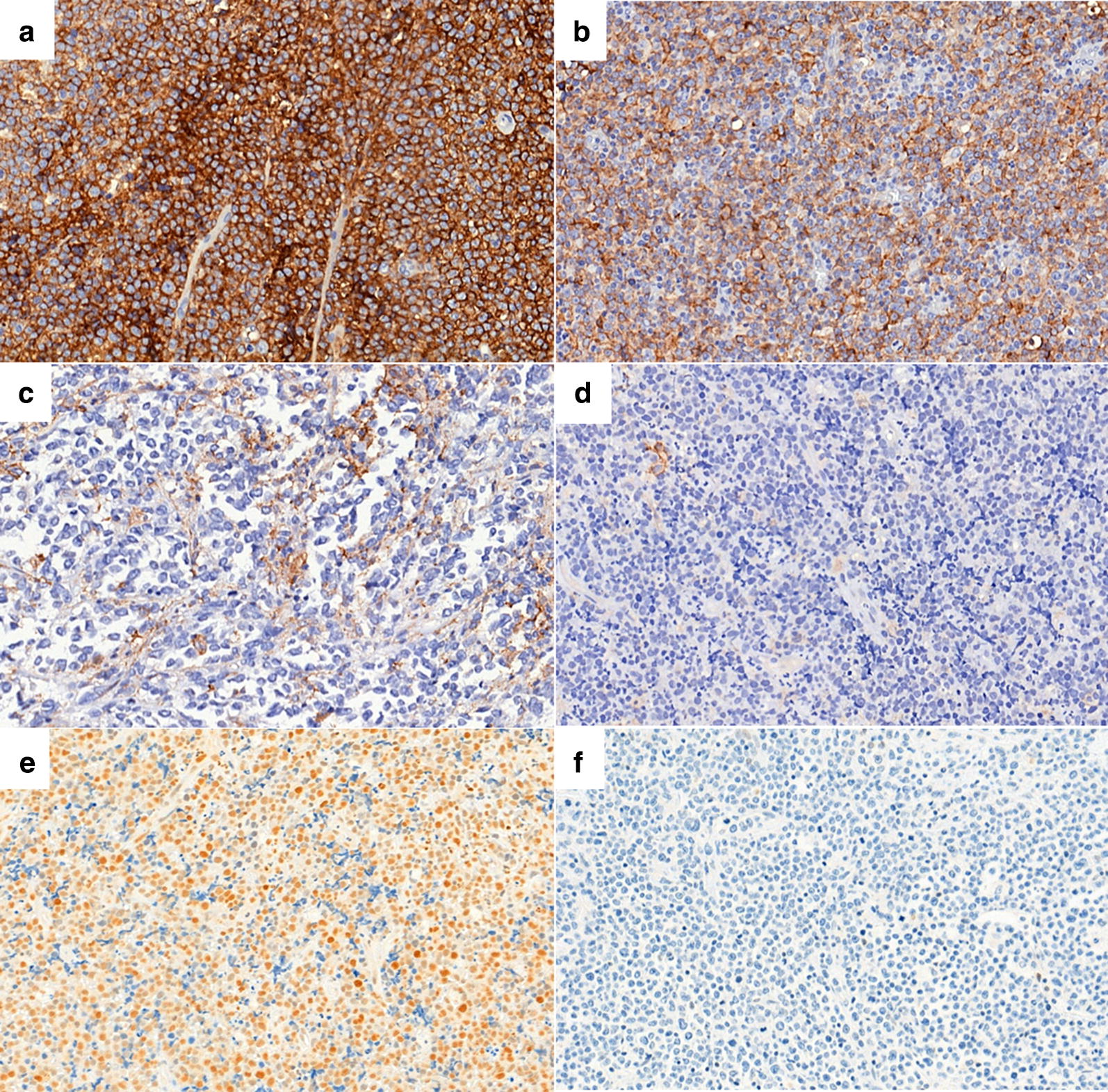
Immunohistochemical expression patterns of PD-L1 and pSTAT3 in diffuse large B cell lymphoma. PD-L1 is expressed in both tumor and immune cells (**a**), only in tumor (**b**) or only in immune cells (**c**). PD-L1 is not expressed in either tumor cells or immune cells (**d**). Tumor cell nuclei are positive for pSTAT3 (**e**), compared to a pSTAT3-negative case (**f**) (×400 magnification)

**Fig. 2:**
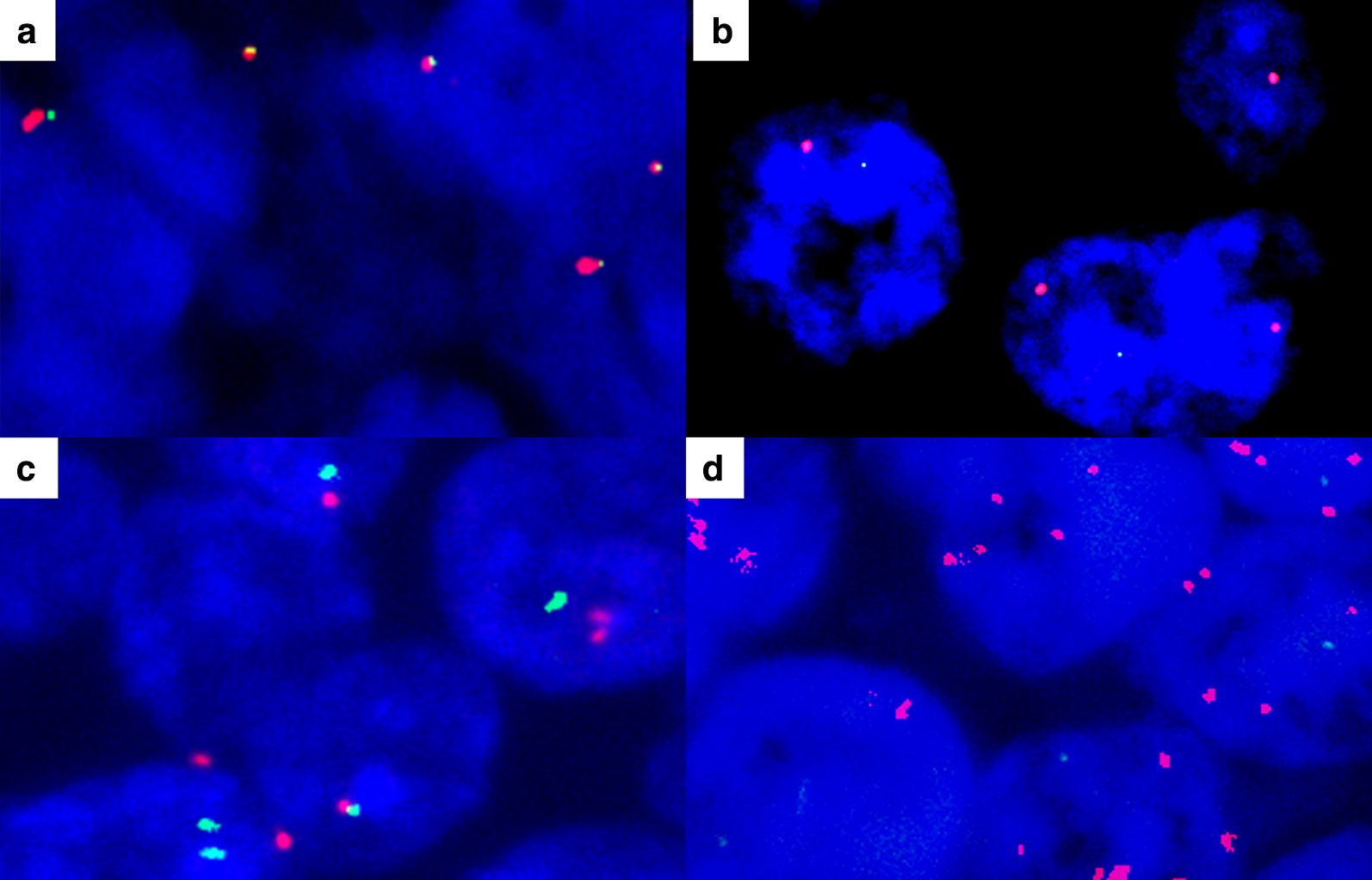
Fluorescence in situ hybridization analysis of PD-L1 gene in diffuse large B cell lymphoma. By using dual-color orange/green break-apart probe, in contrast to non-split fused yellow signals showing no translocation (**a**), separate orange and green signals indicate translocation of PD-L1 gene (**b**). Copy number analysis probes containing orange (PD-L1 gene) and green (chromosome 9) signals show nearly 1:1 ratio of orange to green signals, indicating no increase of copy number of PD-L1 gene (**c**). In contrast, orange signals are amplified, compared to green signals (**d**)

We investigated the frequencies of PD-L1 expression, PD-L1 gene alteration, i.e., translocation, copy number gain and/or amplification, and pSTAT3 expression in tumor cell nuclei according to clinicopathologic parameters in DLBCL patients (Table 2). The PD-L1 expression was interpreted in two aspects as expression in tumor cells (PD-L1t) and expression in immune cells (PD-L1i). The prevalence of PD-L1 expression was 21% (23/107) in tumor cells and 36% (38/107) in non-malignant immune cells. All cases with PD-L1 gene alteration (PD-L1 GA+) expressed PD-L1 protein in tumor cells (PD-L1t+), and accounted for 10% (10/102) of DLBCLs which includes translocation in 6% (6/102) and gain/amplification in 4% (4/102). Nuclear expression of pSTAT3 (> 40% cutoff) was observed in 41% (44/107) of DLBCLs (Figs. 1 and 2).

**Table 2:** Clinicopathologic characteristics according to protein expression and genetic alteration of PD-L1 and STAT3 activation in EBV-negative diffuse large B cell lymphoma patients

Clinicopathologic characteristics	PD-L1 expression in tumor cells (PD-L1t)			PD-L1 expression in immune cells (PD-L1i)			PD-L1 genetic alteration in tumor cells (PD-L1 GA)			pSTAT3^a^ expression in tumor cells		
	PD-L1t−	PD-L1t+	p value	PD-L1i−	PD-L1i+	p value	PD-L1 GA−	PD-L1 GA+	p value	pSTAT3− (< 40%)	pSTAT3+ (> 40%)	p value
Age												
≤ 60	40 (48%)	13 (57%)	0.449^#^	38 (55%)	15 (39%)	0.122^#^	43 (47%)	7 (70%)	0.196^†^	34 (54%)	19 (43%)	0.272^#^
> 60	44 (52%)	10 (43%)		31 (45%)	23 (61%)		49 (53%)	3 (30%)		29 (46%)	25 (57%)	
Sex												
Male	49 (58%)	12 (52%)	0.597^#^	40 (58%)	21 (55%)	0.787^#^	52 (57%)	6 (60%)	1.000^†^	33 (52%)	28 (64%)	0.247^#^
Female	35 (42%)	11 (48%)		29 (42%)	17 (45%)		40 (43%)	4 (40%)		30 (48%)	16 (36%)	
Primary site												
Nodal	44 (52%)	10 (43%)	0.449^#^	33 (48%)	21 (55%)	0.462^#^	50 (54%)	2 (20%)	0.049^†^	27 (43%)	27 (61%)	0.060^#^
Extranodal	40 (48%)	13 (57%)		36 (52%)	17 (45%)		42 (46%)	8 (80%)		36 (57%)	17 (39%)	
B symptoms												
Absent	68 (81%)	17 (74%)	0.561^†^	55 (80%)	30 (79%)	0.926^#^	77 (84%)	6 (60%)	0.087^†^	48 (76%)	37 (84%)	0.320^#^
Present	16 (19%)	6 (26%)		14 (20%)	8 (21%)		15 (16%)	4 (40%)		15 (24%)	7 (16%)	
ECOG PS												
< 2	77 (92%)	20 (87%)	0.445^†^	61 (88%)	36 (95%)	0.489^†^	85 (92%)	8 (80%)	0.214^†^	56 (89%)	41 (93%)	0.520^†^
≥ 2	7 (8%)	3 (13%)		8 (12%)	2 (5%)		7 (8%)	2 (20%)		7 (11%)	3 (7%)	
Serum lactate dehydrogenase^a^												
Normal	45 (56%)	9 (39%)	0.164^#^	34 (52%)	20 (53%)	0.913^#^	47 (51%)	5 (50%)	1.000^†^	32 (52%)	22 (49%)	0.896^#^
Elevated	36 (44%)	14 (61%)		32 (48%)	18 (47%)		42 (49%)	5 (50%)		29 (48%)	21 (51%)	
No. of extranodal sites												
< 2	65 (77%)	16 (70%)	0.439^#^	54 (78%)	27 (71%)	0.405^#^	71 (78%)	5 (50%)	0.118^†^	51 (81%)	30 (68%)	0.130^#^
≥ 2	19 (23%)	7 (30%)		15 (22%)	11 (29%)		21 (22%)	5 (50%)		12 (19%)	14 (32%)	
Ann Arbor stage												
I–II	45 (54%)	11 (48%)	0.625^#^	38 (55%)	18 (47%)	0.445^#^	50 (%)	5 (50%)	1.000^†^	37 (59%)	19 (43%)	0.113^#^
III–IV	39 (46%)	12 (52%)		31 (45%)	20 (53%)		42 (%)	5 (50%)		26 (41%)	25 (57%)	
International prognostic index												
0–2	57 (68%)	14 (61%)	0.530^#^	47 (68%)	24 (63%)	0.603^#^	64 (70%)	5 (50%)	0.286^†^	44 (70%)	27 (61%)	0.361^#^
3–5	27 (32%)	9 (39%)		22 (32%)	14 (37%)		28 (30%)	5 (50%)		19 (30%)	17 (39%)	
Bone marrow involvement^a^												
Absent	66 (87%)	18 (82%)	0.553^†^	54 (86%)	30 (86%)	1.000	76 (89%)	7 (70%)	0.283^†^	51 (88%)	33 (83%)	0.450^#^
Present	10 (13%)	4 (18%)		9 (14%)	5 (14%)		9 (11%)	2 (20%)		7 (12%)	7 (17%)	
Bulky mass (cm)												
< 10	78 (93%)	20 (87%)	0.401^†^	64 (93%)	34 (89%)	0.718^†^	84 (91%)	9 (90%)	1.000^†^	56 (89%)	42 (95%)	0.302^†^
≥ 10	6 (7%)	3 (13%)		5 (7%)	4 (11%)		8 (9%)	1 (10%)		7 (11%)	2 (5%)	
Hans classification^a^												
GCB	26 (34%)	1 (5%)	0.006^†^	20 (32%)	7 (19%)	0.186	26 (30%)	0 (0%)	0.058^†^	20 (35%)	7 (17%)	0.042^#^
Non-GCB	51 (66%)	21 (95%)		43 (68%)	29 (81%)		61 (70%)	10 (100%)		37 (65%)	35 (83%)	
BCL2 expression^a^												
Negative	33 (40%)	8 (35%)	0.665^#^	31 (46%)	10 (26%)	0.051^#^	35 (38%)	2 (20%)	0.321^†^	31 (50%)	10 (23%)	0.004^#^
Positive	50 (60%)	15 (65%)		37 (54%)	28 (74%)		57 (62%)	8 (80%)		31 (50%)	34 (77%)	
BCL6 expression												
Negative	43 (51%)	17 (74%)	0.052^#^	36 (52%)	24 (63%)	0.273^#^	46 (50%)	9 (90%)	0.019^†^	39 (62%)	21 (48%)	0.146^#^
Positive	41 (49%)	6 (26%)		33 (48%)	14 (37%)		46 (50%)	1 (10%)		24 (38%)	23 (52%)	
CD10 expression												
Negative	69 (82%)	23 (100%)	0.037^†^	57 (83%)	35 (92%)	0.248^†^	77 (84%)	10 (100%)	0.351^†^	52 (83%)	40 (91%)	0.268^†^
Positive	15 (18%)	0 (0%)		12 (17%)	3 (8%)		15 (16%)	0 (0%)		11 (17%)	4 (9%)	
MUM1 expression												
Negative	36 (43%)	5 (22%)	0.065^#^	28 (41%)	13 (34%)	0.517^#^	35 (38%)	1 (10%)	0.094^†^	31 (49%)	10 (23%)	0.006^#^
Positive	48 (57%)	18 (78%)		41 (59%)	25 (66%)		57 (62%)	9 (90%)		32 (51%)	34 (77%)	
Total N (%)	84 (100%)	23 (100%)		69 (100%)	38 (100%)		92 (100%)	10 (100%)		63 (100%)	44 (100%)	

NA: not applicable; ECOG PS: the Eastern Cooperative Group Performance Status; GCB: germinal center B-cell like; R-CHOP: rituximab with cyclophosphamide–doxorubicin–vincristine–prednisone; GI: gastrointestinal; GA: genetic alteration; IHC: immunohistochemistry; SD: standard deviation

^a^These variables excluded unclassifiable (or unknown) cases

p values were calculated by using Fisher’s exact test (2-sided)^†^ or Pearson’s Chi square test (2-sided)^#^

In the analysis with clinicopathologic variables (Table 2), PD-L1 GA+ was more frequent in primary extranodal DLBCLs than nodal cases (p = 0.049), and all PD-L1 GA+ cases (n = 10) belonged to non-GCB subtype according to Hans algorithm, while not reaching statistical significance (p = 0.058). As for protein expression, non-GCB subtype also showed more frequent PD-L1 expression (PD-L1t+), as well as pSTAT3 expression, in tumor cells than GCB subtype (p = 0.006 and p = 0.042, respectively). In the analysis with Bcl-2 and each component of Hans algorithm, PD-L1 GA and PD-L1t was significantly or marginally associated with lack of expression of Bcl-6 and/or CD10 (p = 0.019 for PD-L1 GA vs. Bcl-6; p = 0.052 for PD-L1t vs Bcl-6; p = 0.037 for PD-L1t vs CD10), while pSTAT3 expression was mainly related with MUM1 expression (p = 0.006). Of note, PD-L1i was marginally associated with Bcl-2 expression in tumor cells (p = 0.051). Taken together, PD-L1 and/or pSTAT3 signaling pathways are frequently activated in non-GCB subtype or extranodal DLBCLs.

### Association within PD-L1/pSTAT3-related markers

**Fig. 3:**
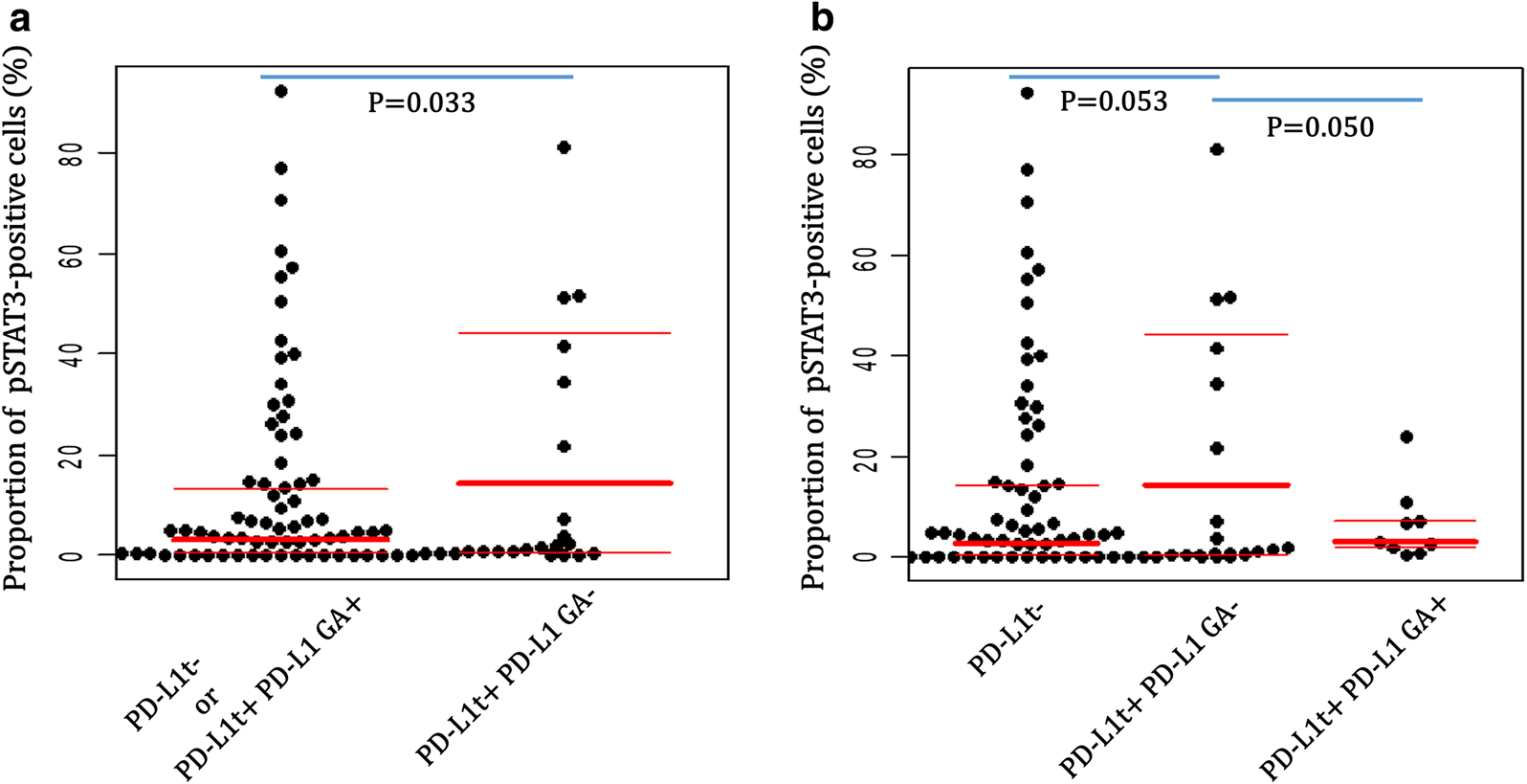
Dot plots for proportions of pSTAT3-positive tumor cells according to PD-L1 protein expression in tumor cells and gene alteration status in diffuse large B cell lymphoma. PD-L1t+ PD-L1 GA− cases have higher proportions (%) of pSTAT3-positive tumor cells than the rest cases (PD-L1t− or PD-L1t+ PD-L1 GA+) (**a**), and this tendency was preserved in comparison of each subset (PD-L1t+ PD-L1 GA− vs. PD-L1t−; PD-L1t+ PD-L1 GA− vs. PD-L1t+ PD-L1 GA+) (**b**)

To clarify the associations between PD-L1 gene/protein status and pSTAT3 expression in DLBCLs, we next performed correlation analysis among PD-L1/pSTAT3-related markers including PD-L1t, PD-L1i, PD-L1 GA and pSTAT3 expression with dichotomized variable by 40% cutoff and continuous variable (%) (Table 3). To effectively recognize associations between PD-L1-related markers and pSTAT3, pSTAT3 was analyzed as two different modes of variables: (1) a digitally counted proportional variable (%) of pSTAT3-positive tumor cells and (2) a conventionally interpreted dichotomized variable with 40% cutoff value. In this way, PD-L1t was positively correlated with PD-L1i, PD-L1 translocation and PD-L1 gain/amplification, while PD-L1i was associated only with PD-L1 translocation. Notably, tumor cells of DLBCLs with PD-L1 expression but no PD-L1 gene alteration (PD-L1t+ PD-L1 GA−) had a higher proportion (%) of pSTAT3-positive tumor cells than the rest of the subset (PD-L1t− or PD-L1t+ PD-L1 GA+) (p = 0.033; Fig. 3a), and in comparison to both PD-L1t− subset (p = 0.053) and PD-L1t+ PD-L1 GA+ subset (p = 0.050) with marginal significance as well (Fig. 3b). These results suggest that PD-L1 protein expression in DLBCL tumor cells lacking intrinsic PD-L1 gene activation mechanism, i.e., translocation and gain/amplification, may be induced by STAT3-mediated signaling pathway.

**Table 3:** Associations between PD-L1, pSTAT3 and other clinicopathologic factors in diffuse large B cell lymphoma (n = 107)

	PD-L1i	PD-L1 translocation (A)	PD-L1 gain/amplification (B)	PD-L1 gene alteration (A + B)	PD-L1 non-genetic expression^b^	pSTAT3^a^ (continuous)	pSTAT3 (> 40%)
PD-L1t	R = 0.325 p = 0.001	R = 0.477 p < 0.001	R = 0.386 p < 0.001	R = 0.629 p < 0.001	R = 0.698 p < 0.001	R = 0.079 p = 0.429	R = 0.071 p = 0.466
PD-L1i		R = 0.239 p = 0.015	R = 0.159 p = 0.109	R = 0.291 p = 0.003	R = 0.166 p = 0.092	R = 0.081 p = 0.414	R = 0.015 p = 0.879
PD-L1 translocation (A)			R = − 0.051 p = 0.614	R = 0.758 p < 0.001	R = − 0.090 p = 0.364	R = − 0.049 p = 0.628	R = − 0.037 p = 0.714
PD-L1 gain/amplification (B)				R = 0.613 p < 0.001	R = − 0.073 p = 0.464	R = − 0.117 p = 0.242	R = − 0.072 p = 0.470
PD-L1 gene alteration (A + B)					R = − 0.120 p = 0.228	R = − 0.117 p = 0.245	R = − 0.021 p = 0.835
PD-L1 non-genetic expression^b^						R = 0.205 p = 0.033	R = 0.120 p = 0.224

Correlation coefficients (R) and p values by Pearson’s correlation

^a^ Indicates the proportion (%) of tumor cells expressing pSTAT3 by using digital image analyzer

^b^ Indicates PD-L1t+ cases with no alteration (translocation or gain/amplification) of PD-L1 gene

### Associations between PD-L1 expression and gene alteration and primary sites

Frequent gene alteration and expression of PD-L1 were known in DLBCLs arising in certain specific sites such as mediastinum and brain [33, 34], both of which were excluded in our cohort. In our cohort, we further analyzed the PD-L1 according to the primary sites, focusing on gastrointestinal tract, testis and adrenal gland. As shown in Table 4, primary testis cases (n = 14) had a higher frequency of PD-L1 gene alteration (29%; 4/14), mainly translocation (21%; 3/14), than non-testis cases (p = 0.029 for PD-L1 GA; p = 0.031 for PD-L1 translocation). Notably, one of the three adrenal gland cases (33%) had a gain/amplification of PD-L1, though with limited significance due to low incidence (p = 0.112). These data suggest that DLBCLs of specific anatomic sites might have preferential alteration patterns of PD-L1 gene.

**Table 4:** Associations between genetic alteration and protein expression status of PD-L1 and primary sites

Primary site	GI			Testis			Adrenal gland		
	Non-GI	GI	p value	Non-testis	Testis	p value	Non-adrenal	Adrenal	p value
PD-L1t									
Absent	67	17	0.354	74	10	0.494	83	1	0.116
Present	21	2		19	4		21	2	
PD-L1i									
Absent	55	14	0.435	59	10	0.766	68	1	0.287
Present	33	5		34	4		36	2	
PD-L1 translocation (A)									
Absent	80	18	0.587	87	11	0.031	95	3	1.000
Present	6	0		3	3		6	0	
PD-L1 gain/amplification (B)									
Absent	81	19	1.000	87	13	0.444	98	2	0.112
Present	4	0		3	1		3	1	
PD-L1 genetic alteration (A + B)									
Absent	75	18	0.203	83	10	0.029	91	2	0.266
Present	10	0		6	4		9	1	

p values were calculated by using Fisher’s exact test (2-sided)

GI: gastrointestinal; GA: genetic alteration

### Survival analysis

**Fig. 4:**
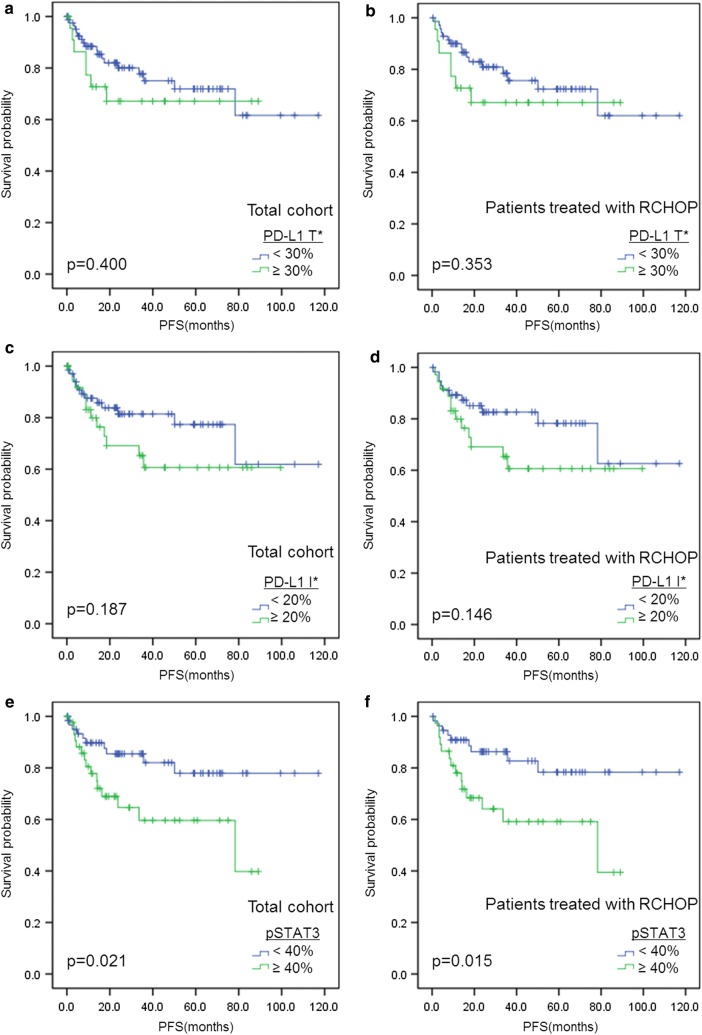
Kaplan–Meier curves of progression-free survival in total and R-CHOP-treated diffuse large B cell lymphoma patients. Survival analysis for progression-free survival (PFS) according to PD-L1t in total cohort (n = 107) (**a**) and R-CHOP cohort (n = 93) (**b**), and according to PD-L1i in total cohort (**c**) and R-CHOP cohort (**d**). Survival analysis for PFS according to pSTAT3 (40% cutoff) in total cohort (**e**) and R-CHOP cohort (**f**). *T: tumor cell; I: immune cell

**Fig. 5:**
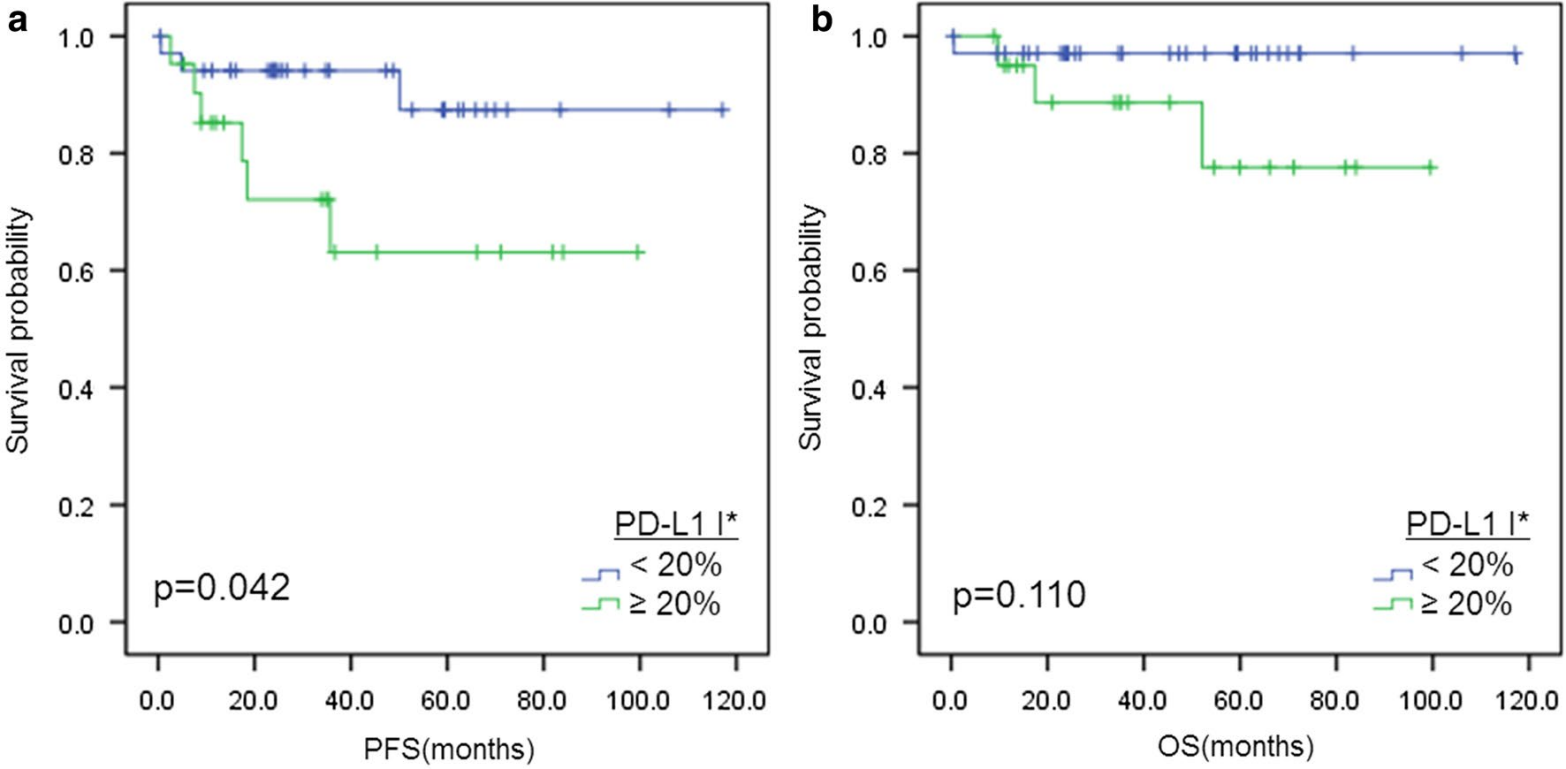
Kaplan–Meier curves of progression-free and overall survival in pSTAT3-negative diffuse large B cell lymphoma patients treated with R-CHOP. Survival analysis for progression-free survival (PFS) according to PD-L1i in pSTAT3-negative R-CHOP treated subset (n = 57) (**a**) and survival analysis for overall survival in pSTAT3-negative R-CHOP treated subset (n = 57) (**b**). * I: non-malignant immune cell

Survival analysis was conducted in total cohort and separately in the subgroup of patients treated with R-CHOP (n = 93; Table 5). No significance was found in clinical outcome with PD-L1 expression alone in either tumor (PD-L1t) or non-malignant immune cells (PD-L1i) (Fig. 4a–d). However, pSTAT3 expression (> 40%) was significantly associated with inferior progression-free survival in the total cohort (p = 0.021; Fig. 4e) and in the R-CHOP-treated group (p = 0.015; Fig. 4f). Multivariate cox regression that followed revealed pSTAT3 to be an independent prognostic factor in both the total cohort (p = 0.017, HR = 2.724) and R-CHOP-treated patients (p = 0.007, HR = 3.510). Other significant prognostic factors in this multivariate analysis were IPI (p = 0.001, HR = 4.910 in total cohort; p = 0.002, HR = 4.823 in the R-CHOP treated group), Eastern Cooperative Group Performance Status (p = 0.010, HR = 3.717 in the total cohort; p = 0.031, HR = 3.286 in R-CHOP treated group) and Bulky mass (p = 0.010, HR = 4.664 in the R-CHOP treated group). When the R-CHOP cohort (n = 93) was divided into pSTAT3-negative (n = 56) and pSTAT3-positive subgroups (n = 37), PD-L1 expression of non-malignant immune cells (PD-L1i) correlated with poor progression-free survival in pSTAT3-negative patients who received R-CHOP regimen by log-rank test in Kaplan–Meier analysis (p = 0.042; Fig. 5). It did not, however, turn out to be an independent prognostic factor (p = 0.089; Table 6), while ECOG PS was the only significant prognostic factor (p < 0.001, HR = 21.553). Analysis with overall survival revealed no significant results (Additional file 1: Figure S1).

**Table 5:** Survival analysis with progression-free survival in total cohort and in patients treated with rituximab plus cyclophosphamide, doxorubicin, vincristine and prednisone (n = 93)

Clinicopathologic variables	Total cohort			Patients treated with R-CHOP		
	Univariate	Multivariate		Univariate	Multivariate	
	p value	p value	HR (95% CI)	p value	p value	HR (95% CI)
Age						
> 60 vs. ≤ 60	0.035	0.181		0.074		
Sex						
Male vs. female	0.544			0.661		
ECOG PS						
≥ 2 vs. < 2	<0.001	0.010	3.717 (1.371–10.080)	< 0.001	0.031	3.286 (1.116–9.677)
B symptoms						
Present vs. absent	0.049	0.807		0.112		
Serum LDH						
Elevated vs. normal	0.003	0.328		0.033	0.319	
IPI						
3–5 vs. 0–2	< 0.001	0.001	4.910 (1.888–12.767)	< 0.001	0.002	4.823 (1.763–13.191)
Primary site						
Extranodal vs. nodal	0.488			0.523		
Extranodal site						
≥ 2 vs. < 2	< 0.001	0.649		0.001	0.978	
Bone marrow involvement						
Present vs. absent	0.308			0.359		
Bulky mass						
≥ 10 cm vs. < 10 cm	0.136			0.048	0.010	4.664 (1.449–15.017)
Ann Arbor stage						
III–IV vs. I–II	< 0.001	0.682		< 0.001	0.812	
Hans classification						
Non-GCB vs. GCB	0.075			0.135		
Treatment regimen						
RCHOP vs. other	0.676			–		
BCL2 expression						
Positive vs. negative	0.391			0.699		
BCL6 expression						
Positive vs. negative	0.247			0.545		
CD10 expression						
Positive vs. negative	0.543			0.778		
MUM1 expression						
Positive vs. negative	0.138			0.211		
PD-L1 tumor cell expression						
Positive vs. negative	0.400			0.353		
PD-L1 immune cell expression						
Positive vs. negative	0.187			0.146		
PD-L1 gene alteration						
Present vs. absent	0.900			0.871		
pSTAT3 expression (> 40%)						
Positive vs. negative	0.021	0.017	2.724 (1.196–6.204)	0.015	0.007	3.510 (1.418–8.692)

PD-L1: programmed cell death-ligand 1; pSTAT3: phosphorylated signal transducer and activator of transcription 3; ECOG PS: the Eastern Cooperative Group Performance Status; GCB: germinal center B-cell like; R-CHOP: rituximab plus cyclophosphamide, doxorubicin, vincristine and prednisone

**Table 6:** Survival analysis with progression-free survival in patients treated with rituximab plus cyclophosphamide, doxorubicin, vincristine and prednisone (n = 56)

Clinicopathologic variables	Univariate	Multivariate	
	p value	p value	HR (95% CI)
Age			
> 60 vs. ≤ 60	0.158		
Sex			
Male vs. female	0.702		
ECOG PS			
≥ 2 vs. < 2	< 0.001	< 0.001	21.553 (4.682–99.208)
B symptoms			
Present vs. absent	0.099		
Serum LDH			
Elevated vs. normal	0.189		
IPI			
3–5 vs. 0–2	0.002	0.238	
Primary site			
Extranodal vs. nodal	0.204		
Extranodal site			
≥ 2 vs. < 2	< 0.001	0.167	
Bone marrow involvement			
Present vs. absent	0.476		
Bulky mass			
≥ 10 cm vs. < 10 cm	0.279		
Ann Arbor stage			
III–IV vs. I–II	0.037	0.610	
Hans classification			
Non-GCB vs. GCB	0.503		
BCL2 expression			
Positive vs. negative	0.983		
BCL6 expression			
Positive vs. negative	0.600		
CD10 expression			
Positive vs. negative	0.600		
MUM1 expression			
Positive vs. negative	0.409		
PD-L1 tumor cell expression			
Positive vs. negative	0.209		
PD-L1 immune cell expression			
Positive vs. negative	0.042	0.089	
PD0L1 gene alteration			
Present vs. absent	0.285		

PD-L1: programmed cell death-ligand 1; pSTAT3: phosphorylated signal transducer and activator of transcription 3; ECOG PS: the Eastern Cooperative Group Performance Status; GCB: germinal center B-cell like; R-CHOP: rituximab with cyclophosphamide, doxorubicin, vincristine and prednisone

## Discussion

The roles of PD-L1 expression and gene alteration are relatively unclear in EBV-negative DLBCLs, compared to EBV-positive DLBCLs. In this study, we observed that (1) immunopositivity for PD-L1 was 21% in tumor cells which included all the cases of PD-L1 gene alteration that accounts for 10%, and 36% in immune cells, while pSTAT3 positivity was 41% in tumor cells, (2) the non-GCB subtype showed higher PD-L1 and pSTAT3 tumor cell expression and a tendency for PD-L1 gene alteration, (3) PD-L1 expression without gene alteration correlated with pSTAT3 expression, (4) pSTAT3 expression independently predicted shorter PFS in total cohort and R-CHOP-treated group and (5) PD-L1 expression in immune cells correlated with shorter PFS in pSTAT3-negative R-CHOP-treated subset, while influenced by ECOG PS.

Research on PD-L1 in DLBCL has started rather recently and the evaluation methods or standards of PD-L1 immunohistochemistry in DLBCL has not yet met consensus. Studies vary in the PD-L1 antibodies used, the method of evaluation (manual or digital) and the cutoff values to determine positivity. The prevalence of PD-L1 expression ranges greatly from 10.5 to 61.1% in tumor cells and 15.3 to 30% in non-malignant immune cells [4–6, 35, 36], which are similar to our observation.

In the present study, tumor cell expression of PD-L1, but not the non-malignant immune cell expression, significantly associated with non-GCB DLBCL. This is in concordance with previous studies [6–8]. Siddiqi and colleagues provided more concrete evidence of the association between PD-L1 expression and Hans algorithm classification by conducting next generation sequencing on primary DLBCL specimens [9]. In their study, the positive correlation of PD-L1 expression and mutations associated with non-GCB DLBCL such as FAT2 contrasted with the negative correlation of mutations associated with GCB DLBCL such as BCL2, FOXO1 and KMT2D. Considering that PD-L1 gene tended to be frequently altered in non-GCB or extranodal DLBCLs, PD-L1 gene alteration may be one of the important genetic features of non-GCB DLBCLs, and these findings may provide an insight into the understanding of the pathogenesis of non-GCB DLBCLs.

Among various extranodal DLBCLs, several site-specific variants or distinct entities have been described [29]. Primary testicular DLBCLs are well-known to have unique clinicopathologic features with frequent PD-L1 gene alteration [34]. In accordance with these reports, we observed frequent PD-L1 gene alteration of testicular DLBCLs in our cohort as well, and translocation was the predominant form of alteration. Primary adrenal DLBCL is another very rare extranodal subset with unique clinical features [37]. However, the underlying genetic alteration is not well characterized. In our observation, one of the three adrenal DLBCL harbored PD-L1 gain/amplification, although the clinical significance could not yet be determined. This finding might provide a clue to adrenal lymphomagenesis and lead to further investigation of PD-L1 gene alteration in adrenal DLBCLs.

The 41% positivity of pSTAT3 immunohistochemistry of this study is in agreement with most reports [27, 38–40]. Increased expression of pSTAT3 also showed significant association with non-GCB DLBCL. Constitutive activation of STAT3 in non-GCB DLBCL has been investigated more in depth than PD-L1. Higher levels of STAT3 mRNA was detected in the non-GCB subtype than the GCB subtype using DLBCL cell lines [22, 28] and pSTAT3 appeared to bind to promoters of different genes in each subtype, wherein genes regulating cell proliferation and survival are mostly upregulated in non-GCB DLBCL [28]. The preferential activation of STAT3 in non-GCB DLBCL may in part be explained by Bcl-6-induced down-regulation of STAT3 in non-GCB DLBCL cells [23]. It is of note that Bcl-6 is a typical GCB marker and its expression level is generally low in non-GCB DLBCL, possibly leading to STAT3 activation.

Activated PD-1/PD-L1 axis plays the role of tumor evasion from host tumor-specific T-cell immunity. Conceptually, the activation mechanism of PD-L1 gene may be divided into the intrinsic alteration of PD-L1 gene and activation of its upstream signaling pathway, which may contain STAT3 signaling [17]. This might be supported by our observation that the frequency of PD-L1 protein expression (21%) is higher than PD-L1 gene alteration (10%). In the present study, cases with no alteration of PD-L1 gene (PD-L1t+ PD-L1 GA−) tended to have higher proportions (%) of pSTAT3-positive cells than PD-L1t− subset and PD-L1t+ PD-L1 GA+ subset. This finding suggests that STAT3-mediated PD-L1 expression and genetically activated PD-L1 expression might partly be mutually exclusive, and STAT3-mediated signaling might be an alternative mechanism for PD-L1 expression, which remains to be clarified further.

Our finding in survival analysis for pSTAT3 expression as an independent prognostic factor for shorter progression-free survival is consistent with the previous reports in DLBCLs [19, 25, 39, 40]. Huang and colleagues [40] took a step further in taking DLBCL cell lines to gene expression profiling analysis and found an 11-gene STAT3 signature including *CD48*, *IRF1* and *IL10*, which correlated well with inferior clinical outcomes. In an experimental animal model, microenvironmental immature dendritic cells coproducing IL-10 and PD-L1 enhanced anti-tumor immune reaction [41]. This finding suggests the cooperative immunosuppressive role of IL-10 and PD-L1, which may prevail in the STAT3-skewed microenvironment of non-GCB DLBCLs. Considering that IL-10 is also produced by B cells via Toll-like receptor/MyD88/STAT3 pathway in immune reaction [42], the mechanism of interplay between neoplastic B cells and non-malignant immune cells with activated STAT3- and PD-L1-related signaling in the milieu of IL-10 may be more complex than solid tumor models. In this context, the effects of PD-L1 on clinical outcome need to be carefully analyzed with distinctive interpretation of its expression on tumor cells and immune cells with consideration of activation status of the STAT3-related signaling pathway.

Few have investigated the prognostic value of PD-L1 in DLBCL and the results are controversial. Kiyasu and colleagues [6] reported that PD-L1 expression of DLBCL tumor cells was associated with poor clinical outcome whereas that of non-malignant stromal cells showed no significant difference in prognosis. Siddiqi’s group [9] also found PD-L1 tumor cell expression to be associated with inferior survival while Kwon and colleagues [35] reported no significant association to clinical outcome in DLBCL. In the present study, though PD-L1 tumor cell expression had no prognostic significance, immune cell expression of PD-L1 was associated with poor outcome in the pSTAT3-negative R-CHOP-treated subset in univariate analysis. It is not clear why this prognostic effect of PD-L1 expressing immune cell was observed in this subset. One explanation might be that paucity of STAT3-related signature could make the immune microenvironment more dependent on PD-L1 signaling. Furthermore, the pSTAT3-positive subset may have robust STAT3-driven survival signaling of tumor cells that can override the effect of PD-L1-mediated immune evasion [23, 30, 43]. In another point of view, the prognostic role of immune cell PD-L1 may be related with tumoral Bcl-2 expression in our study, where both markers of different cell types had marginal association. The intrinsic mechanism of cancer cell may influence anti-tumor immunity [44, 45], where Bcl-2 protein may act as a tumor-associated antigen [46], although further studies are required for support. In this context, proper isolation of clinicopathologic subsets may provide chances for efficient therapeutic application in targeting PD-L1 signaling in DLBCL patients.

## Conclusion

Our study revealed the association between gene alteration and protein expression of PD-L1 and pSTAT3 expression, both of which constituted features of non-GCB DLBCLs. We also observed that DLBCL patients with pSTAT3-positive tumors had an independently inferior clinical outcome, while in those with pSTAT3-negative tumors, PD-L1 immune cell expression was predictive of poor prognosis. These findings may open another potential immunotherapeutic strategy for the treatment of DLBCL.

## Additional file

**Additional file 1: Figure S1.** Kaplan–Meier curves of overall survival in total and R-CHOP-treated diffuse large B cell lymphoma patients. *T, tumor cell; I, immune cell.

## Abbreviations

DLBCL: diffuse large B cell lymphoma
PD-L1: programmed cell death ligand 1
pSTAT3: phosphorylated signal transducer and activator of transcription 3
GCB: germinal center B cell like
R-CHOP: rituximab combined with cyclophosphamide–doxorubicin–vincristine–prednisone
EBV: Epstein–Barr virus
PD-L1t: PD-L1 expression in tumor cells
PD-L1i: PD-L1 expression in immune cells
PD-L1 GA: PD-L1 gene alteration
