# Supplementary figures and images for: Clinicopathologic implication of PD-L1 and phosphorylated STAT3 expression in diffuse large B cell lymphoma
Source: J Transl Med. 2018 Nov 20;16:320. doi: 10.1186/s12967-018-1689-y (PMC6245852; doi:10.1186/s12967-018-1689-y)

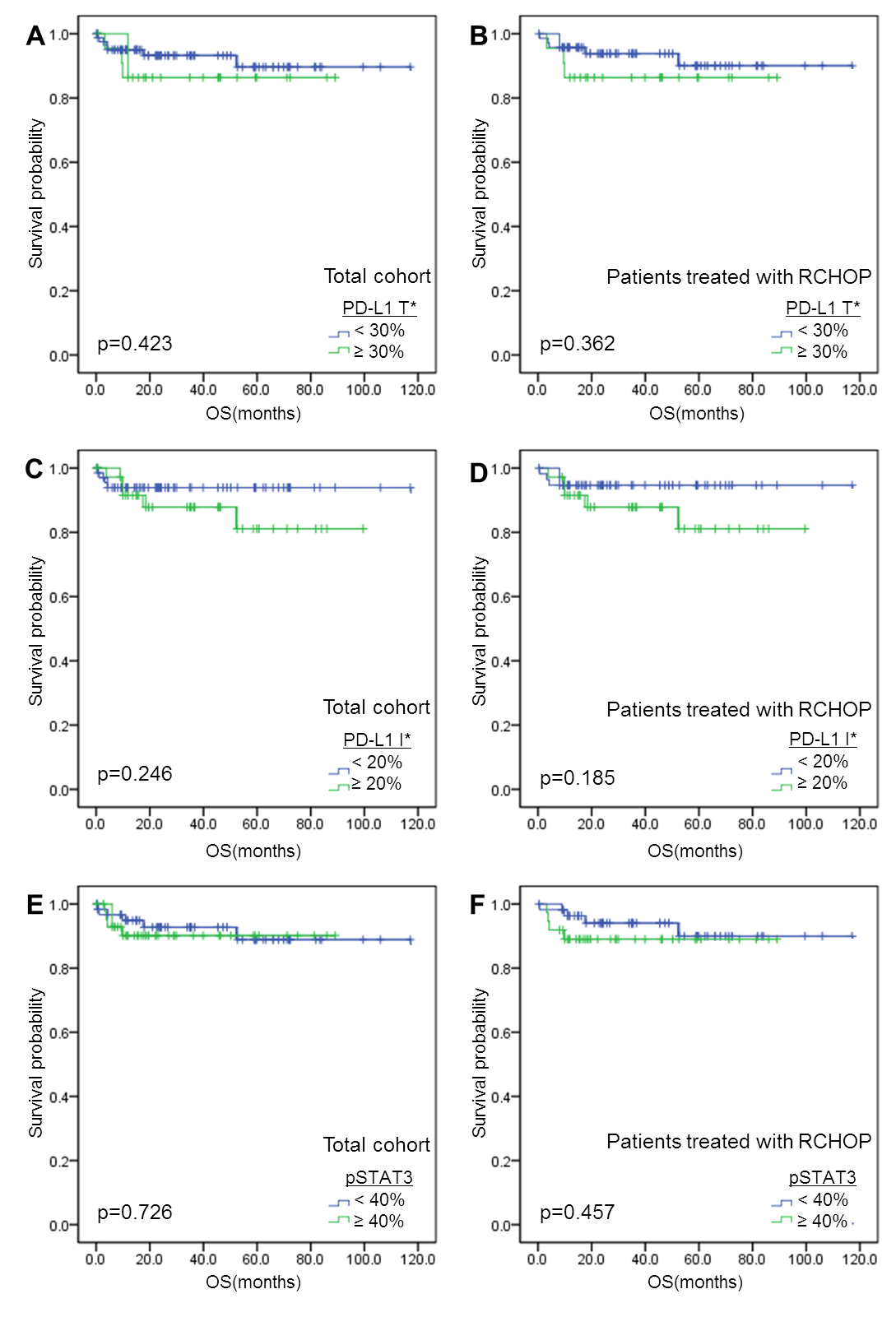

Supplement: Supplementary file 1 — Additional file 1: Figure S1. Kaplan–Meier curves of overall survival in total and R-CHOP-treated diffuse large B cell lymphoma patients. *T, tumor cell; I, immune cell. [file 12967_2018_1689_MOESM1_ESM.tif]
